# Student-Reported School Drinking Fountain Availability by Youth Characteristics and State Plumbing Codes

**DOI:** 10.5888/pcd11.130314

**Published:** 2014-04-17

**Authors:** Stephen J. Onufrak, Sohyun Park, Cara Wilking

**Affiliations:** Author Affiliations: Sohyun Park, Centers for Disease Control and Prevention, Atlanta, Georgia; Cara Wilking, Northeastern University School of Law, Boston, Massachusetts.

## Abstract

**Introduction:**

Caloric intake among children could be reduced if sugar-sweetened beverages were replaced by plain water. School drinking water infrastructure is dictated in part by state plumbing codes, which generally require a minimum ratio of drinking fountains to students. Actual availability of drinking fountains in schools and how availability differs according to plumbing codes is unknown.

**Methods:**

We abstracted state plumbing code data and used the 2010 YouthStyles survey data from 1,196 youth aged 9 through 18 years from 47 states. We assessed youth-reported school drinking fountain or dispenser availability and differences in availability according to state plumbing codes, sociodemographic characteristics, and area-level characteristics.

**Results:**

Overall, 57.3% of youth reported that drinking fountains or dispensers in their schools were widely available, 40.1% reported there were only a few, and 2.6% reported that there were no working fountains. Reported fountain availability differed significantly (*P* < .01) by race/ethnicity, census region, the fountain to student ratio specified in plumbing codes, and whether plumbing codes allowed substitution of nonplumbed water sources for plumbed fountains. “Widely available” fountain access ranged from 45.7% in the West to 65.4% in the Midwest and was less common where state plumbing codes required 1 fountain per more than 100 students (45.4%) compared with 1 fountain per 100 students (60.1%) or 1 fountain per fewer than 100 students (57.6%).

**Conclusion:**

Interventions designed to increase consumption of water may want to consider the role of plumbing codes in availability of school drinking fountains.

## Introduction

Americans can limit excess caloric intake and weight gain by drinking water and other beverages with few or no calories (1) and caloric intake could be reduced among children if sugar-sweetened beverages were replaced by plain water (2). However, more than 50% of US high school students drank 3 or fewer glasses or bottles of water per day in 2010 (3) and 19% drank 2 or more cans, glasses, or bottles of soda per day in 2011 (4). Adequate water consumption also prevents dehydration, which is associated with poor cognitive function in youth (5).

Providing greater access to free drinking water in schools is a strategy to support healthy beverage intake among youth (6,7). The Institute of Medicine recommended that free drinking water be available throughout the school day to students (8). The Healthy, Hunger-Free Kids Act of 2010 requires that schools participating in federal meal programs make free drinking water available to children during meals in the cafeteria (9). Outside of the cafeteria, access to free drinking water is regulated by state and local plumbing codes. These codes can specify the number of drinking fountains, the placement of fountains, and whether or not some or all of the plumbed fountains may be replaced by nonplumbed water dispensers. Plumbing codes are generally enforced during initial school construction or when schools undergo major renovation. Furthermore, it is unknown how plumbing codes might affect water availability over the life of a school as plumbing fixtures break down and temporary mobile classrooms are added to accommodate student enrollment beyond the original design capacity of school buildings. In 1999, 25% of public schools had plumbing in “less than adequate condition” (10), and in 2005, 33% of all public schools had temporary classroom facilities (11). Therefore, we investigated student-reported access to drinking fountains in schools and whether access differed according to individual and area-level characteristics, including state plumbing codes.

## Methods

### Youth survey data

We used the 2010 YouthStyles survey, which was administered by Synovate, Inc, a market research firm. This national mail survey assessed health-related attitudes and behaviors among children. Participants were selected from children of respondents to the ConsumerStyles survey, a consumer mail survey drawn from a mail panel of 200,000 potential respondents. The sampling design is stratified by region, household income, population density, age, and household size and includes an oversample of low-income and minority participants and households with children to ensure adequate representation of these groups. In 2010, a total of 10,328 people completed the ConsumerStyles survey (response rate, 51.6%).

Following survey completion by ConsumerStyles participants, 2,401 YouthStyles surveys were sent to a stratified sample of adults with children aged 9 through 19 years living at home, with instructions that the sampled youth should complete the survey. Of the 2,401 YouthStyles surveys sent, 1,197 surveys were completed, yielding a youth response rate of 49.9%. Respondents were youth from 47 states and the District of Columbia. Alaska, Hawaii, and Wyoming were not represented in the study population. The YouthStyles survey is weighted to match the US Current Population Survey (12). Individuals in the final sample were assigned sample weights according to the age and sex of the child, household size, household income, head of household’s age, and race/ethnicity of the adult included in the ConsumerStyles survey.

There were no statistical differences in YouthStyles responders and nonresponders by parental income, home ownership, household size, or sex of child. However, response rates differed by child age (χ^2^ test, *P* = .001) and parent race/ethnicity (*P* < .001). Higher response rates were seen among high school–aged youth (55%) compared with middle school–aged (50%) and elementary school–aged (48%) youth. Lower response rates were seen among Hispanics (37%) compared with non-Hispanic (NH) whites (55%) and NH blacks (52%).

We excluded from analyses 57 participants because of a missing or invalid response to the question regarding school drinking fountains, resulting in a sample of 1,140 youth. Additional participants were excluded from analyses involving plumbing code variables because their state did not have a code or because the code did not specify a fountain to student ratio or a proportion of fountains that could be replaced by nonplumbed sources. As a result, 62 participants were excluded from analyses of fountain to student ratios and 43 participants were excluded from analyses of nonplumbed water sources, leaving sample sizes of 1,078 and 1,097, respectively. Excluded participants did not differ with regards to age, sex, region, or income but included a higher proportion of Hispanic and “other race” youth.

Participants were asked to rate their agreement with the following statements: “Does your school have working drinking fountains or dispensers that you can drink from?” Response choices available were “Yes, there are many,” “Yes, only are a few,” “There are no water fountains,” “No, none work,” and “I don’t know.” After excluding those youth who responded “I don’t know” (missing or invalid responses) as noted above, 2 categories were created for the water access variable: 1) drinking fountains widely available, and 2) few or no working drinking fountains.

We included in our analysis demographic variables of sex, race/ethnicity (NH white, NH black, Hispanic, or NH Other), age (9–10 y [elementary school], 11–13 y [middle school], and 14–18 y [high school]), and annual household income (<$25,000, $25,000–$59,999, and >$59,999). Cutpoints for annual household income approximately correspond to 100% and 250% income to poverty level ratios for a family of 4.

Respondents were classified by the respondent’s state of residence according to the following 4 US Census regions: Northeast, South, Midwest, and West (13). The size of the Census Bureau–defined metropolitan statistical area in which the participant resided during the survey was classified as either a nonmetropolitan area, a metropolitan or micropolitan area population of fewer than 500,000, or a metropolitan area of 500,000 or more.

### Plumbing code variables

Plumbing code data for each state were obtained through legal research of each state during 2012. First, we consulted online data available from the International Code Council (www.iccsafe.org) and Reed Construction Data (www.reedconstructiondata.com) to determine whether a particular state had adopted a statewide plumbing code. Next, we conducted state-by-state searches using the Westlaw legal database of state administrative codes (www.westlaw.com) to identify codified plumbing code provisions and extract citations. If a model code or other statewide plumbing code was adopted, we determined whether it was applicable to school buildings and whether the code had been modified. We also searched state department of education regulations for the terms “water” and “drinking fountain” and state department of health regulations for “drinking fountain” and “school facilities” in reference to statewide plumbing codes.

With the exception of Arkansas, where the school fountain requirement was repealed 1 month before code abstraction, the codes referenced are the codes that were in effect as of August 2012. If school-specific plumbing provisions differentiated between “existing” and “new” buildings, the fountain requirement for “new” facilities was used. Several states recognize model codes (such as the International Plumbing Code [www.iccsafe.org/cs/PMG/Pages/IPC.aspx]) that are not mandatory but rather optional for local jurisdictions to adopt. Some states have school-specific provisions that allow the use of a local building code or a model building code. In all these cases, the school drinking fountain standard that was contained in a referenced model code was used. Statewide building codes could not be abstracted in Mississippi and Missouri because neither state adopted statewide plumbing codes setting minimum fountain standards.

For each state with available code data, we abstracted the minimum fixture standards for drinking fountains in elementary and secondary school buildings and determined whether schools were allowed to substitute nonplumbed water coolers for plumbed drinking fountains. The required number of fountains per student was classified as 1 fountain per fewer than 100 students, 1 fountain per 100 students, or 1 fountain per more than 100 students. On the basis of the average class size, Louisiana, where code specified “1 per each 3 classrooms with at least one per floor,” was classified as 1 fountain per fewer than 100 students. Plumbing codes for Montana and Oregon only specified 1 fountain per floor and did not specify a required number of fountains per student. On the basis of what was found in codes across states, the percentage of fountains that can be replaced with bottled water coolers was classified in analyses as none, 50%, or 100%.

### Analysis

All analysis was conducted using SAS 9.3 (SAS Institute, Inc, Cary, North Carolina). First, univariate frequencies of perceived school drinking fountain access were estimated. Next, bivariate analysis was performed to assess differences in prevalence of widely available school drinking water access according to age, sex, race/ethnicity, household income, region, metropolitan status, the fountain to student ratio specified in the plumbing code, and whether the plumbing code allowed nonplumbed water dispensers. Because all state plumbing codes that specify fountain to student ratios of 1 fountain per more than 100 students also do not allow substitution of plumbed drinking fountains, and because nearly all observations in the data set corresponding with fountain to student ratios of 1 fountain per more than 100 students occur in the West, it was not possible to include these 3 variables (ie, region, fountain to student ratio, and allowance of nonplumbed water dispensers) in the same model. Therefore, 3 multivariable logistic models were fit after controlling for age, sex, race/ethnicity, household income, and metropolitan status. This analysis was exempt from the Centers for Disease Control and Prevention (CDC) Institutional Review Board process because personal identifiers were not included in the data provided to CDC.

## Results

Overall, 57.3% of respondents reported that school drinking fountains or dispensers were widely available, 40.1% reported there were only a few, and 2.6% reported that there were no working fountains available in their schools. Statewide plumbing codes for school buildings are shown in Table 1. The minimum fountain to student ratio ranged from 1 fountain per 30 students (Arkansas) to 1 fountain per the first 150 students and 1 fountain per each additional 500 students (Washington). The most common ratio was 1 fountain per 100 students (31 states). Regarding nonplumbed water dispensers, 26 states allowed nonplumbed water coolers to be substituted for up to 50% of plumbed fountains, and 5 states allowed substitution of 100% of plumbed fountains.

**Table 1:** Summary Table of Abstracted School Water Fountain Requirements According to State Plumbing Codes, 2012^a^

State	Ratio of Fountains to Students	% of Fountains That Can Be Replaced With Nonplumbed Water Sources
Alabama	1 per 100	50%
Alaska	1 per first 150 and 1 per each 300 thereafter	None
Arizona	1 per 50 (grades K–8); 1 per 100 (grades 9–12)	100%
Arkansas	1 per 30	None
California	1 per first 150 and 1 per each 300 thereafter	None
Colorado	1 per 100	50%
Connecticut	1 per 100	50%
Delaware	1 per 100	50%
Florida	1 per 100	50%
Georgia	1 per 100	50%
Hawaii	1 per 100	50%
Idaho	1 per first 150 and 1 per each 300 thereafter	None
Illinois	1 per 75	100%
Indiana	1 per 75	100%
Iowa	1 per 100	50%
Kansas	1 per 100	50%
Kentucky	1 per 75	100%
Louisiana	1 per each 3 classrooms with at least 1 per floor	None
Maine	1 per first 150 and 1 per each 300 thereafter	None
Maryland	1 per 100	50%
Massachusetts	1 per 75	None
Michigan	1 per 100	50%
Minnesota	1 per 100	50%
Mississippi	No statewide code identified	No statewide code identified
Missouri	No statewide code identified	No statewide code identified
Montana	1 per floor	Individual case-by-case basis
Nebraska	1 per first 150 and 1 per each 300 thereafter	None
Nevada	1 per 100	50%
New Hampshire	1 per 40	None
New Jersey	1 per 100	None
New Mexico	1 per 100	50%
New York	1 per 100	50%
North Carolina	1 per 100	None
North Dakota	1 per 100	100%
Ohio	1 per 100	50%
Oklahoma	1 per 100	50%
Oregon	1 per floor	None
Pennsylvania	1 per 100	50%
Rhode Island	1 per 100	50%
South Carolina	1 per 100	None
South Dakota	1 per first 150 and 1 per each 300 thereafter	None
Tennessee	1 per 100	50%
Texas	1 per 100	50%
Utah	1 per 100	50%
Vermont	1 per 100	50%
Virginia	1 per 100	50%
Washington	1 per first 150 and 1 per each 500 thereafter	None
West Virginia	1 per 100	50%
Wisconsin	1 per 100	50%
Wyoming	1 per 100	None

a Plumbing code data for each state obtained through legal research during 2012 by using online data from the International Code Council (www.iccsafe.org), Reed Construction Data (reedconstructiondata.com), Westlaw legal database of state administrative codes (www.westlaw.com), and state departments of education regulations. With the exception of Arkansas, the codes referenced were the codes in effect as of August 2012.

Perceived school water access differed by race/ethnicity, region, fountain to student ratio specified in the plumbing code, and allowance of nonplumbed water dispensers (Table 2). Widely available fountains or dispensers were reported at the highest frequency by black youth (67.8%) and least frequently by Hispanic youth (50.4%). Regional prevalence of widely available school fountains ranged from 65.4% in the Midwest to 45.7% in the West. Widely available school drinking fountains were reported less frequently where state plumbing codes specified a fountain to student ratio of 1 fountain per more than 100 students (45.4% vs 60.1% for 1 per 100 students and 57.6% for 1 per fewer than 100 students). A greater percentage of students who lived in states where nonplumbed water sources could be substituted for all or half of drinking fountains reported widely available water access (61.0% for 100% substitution allowed and 61.1% for 50% substitution allowed vs 48.5% for no substitution allowed).

**Table 2:** Student-Reported Availability of School Water Fountains or Dispensers by Sociodemographic Characteristics and State Plumbing Codes for Schools^a^

Characteristic (No. of Respondents)	Student-Reported Availability of School Water Fountains or Dispensers^b^		χ^2^ *P* Value
	Few or None	Widely Available	
**Overall N (weighted %)**	487 (42.6%)	653 (57.4%)	NA
**Age, y**			
9–10 (n = 221)	44.4%	55.6%	.45
11–13 (n = 326)	39.8%	60.2%	
14–18 (n = 593)	43.4%	56.6%	
**Sex**			
Male (n = 643)	40.9%	59.1%	.27
Female (n = 497)	44.3%	55.7%	
**Race/ethnicity**			
White (n = 775)	43.0%	57.0%	.009
Black (n = 125)	32.2%	67.8%	
Hispanic (n = 159)	49.6%	50.4%	
Other (n = 81)	44.9%	55.1%	
**Annual household income, $**			
<25,000 (n = 166)	42.8%	57.2%	.55
25,000–59,999 (n = 324)	44.5%	55.5%	
>59,999 (n = 650)	41.2%	58.8%	
**Census region**			
Northeast (n = 227)	48.5%	51.5%	<.001
South (n = 423)	39.4%	60.4%	
Midwest (n = 290)	34.6%	65.4%	
West (n = 200)	54.3%	45.7%	
**Metropolitan statistical area**			
Nonmetropolitan area (n = 233)	43.8%	56.2%	.30
Population <500,000 (n = 204)	37.9%	62.1%	
Population ≥500,000 (n = 703)	43.5%	56.5%	
**Drinking fountains required per student per state plumbing code^c^**			
1 fountain per <100 students (n = 182)	42.5%	57.6%	.0048
1 fountain per 100 students (n = 750)	40.0%	60.1%	
1 fountain per >100 students (n = 146)	54.6%	45.4%	
**Nonplumbed water sources allowed?^d^**			
Nonplumbed water sources may substitute for all fountains (n = 135)	39.0%	61.0%	.0007
Nonplumbed water sources may substitute half of fountains (n = 647)	39.0%	61.1%	
Nonplumbed water sources may not substitute for fountains (n = 315)	51.5%	48.5%	

Abbreviation: NA, not applicable.

a Values are expressed as percentages unless otherwise indicated. Rows may not sum to 100 because of rounding

b Participants responded either “Yes, there are many,” “Yes, only a few,” “No, none work,” or “There are no water fountains” to the question “Does your school have working drinking fountains or dispensers that you can drink from?” “Widely available drinking fountains” correspond with participants reporting “Yes, there are many.”

c Excluded participants in states where there was no code.

d Excluded participants in states where the state code did not specify a fountain to student ratio or a proportion of fountains that could be replaced by nonplumbed sources.

In multivariable analysis, black youth were more likely to report widely available fountains or dispensers compared with white youth (odds ratio [OR], 1.6; 95% confidence interval [CI], 1.1–2.3). Youth living in the Midwest (OR, 2.2; 95% CI 1.5–3.2) and South (OR, 1.6; 95% CI, 1.2–2.3) were significantly more likely to report widely available school drinking fountains than youth in the West. Youth living in states where plumbing codes required 1 fountain per more than 100 students were significantly less likely (OR, 0.6; 95% CI, 0.4–0.9) to report widely available drinking fountains than youth residing where plumbing codes specified 1 fountain per 100 students. Compared with youth residing where nonplumbed drinking fountains were not allowed, youth residing in states where 100% or 50% substitution of plumbed fountains with nonplumbed water dispensers was allowed were significantly more likely to report widely available drinking water fountains or dispensers (OR, 1.7; 95% CI, 1.1–2.6 for 100% substitution allowed; OR, 1.7; 95% CI, 1.3–2.2 for 50% substitution allowed) (Table 3).

**Table 3:** Multivariable Adjusted Associations of Sociodemographic, Area, and State Plumbing Code Characteristics with Student-Reported Widely Available Drinking Fountains or Dispensers at School^a^

Variable	Odds Ratio (95% Confidence Interval)^b^
**Age, y**	
9–10 (n = 221)	1 [Reference]
11–13 (n = 326)	1.2 (0.8–1.7)
14–18 (n = 593)	1.0 (0.8–1.4)
**Sex**	
Male (n = 643)	1 [Reference]
Female (n = 497)	0.9 (0.7–1.1)
**Race/ethnicity**	
White (n = 775)	1 [Reference]
Black (n = 125)	1.6 (1.1–2.3)
Hispanic (n = 159)	0. 9 (0.6–1.2)
Other (n = 81)	1.0 (0.6–1.9)
**Annual household income, $**	
<25,000 (n = 166)	1 [Reference]
25,000–59,999 (n = 324)	0.9 (0.7–1.4)
>59,999 (n = 650)	1.1 (0.8–1.6)
**Census region**	
Northeast (n = 227)	1.2 (0.8–1.8)
South (n = 423)	1.6 (1.2–2.3)
Midwest (n = 290)	2.2 (1.5–3.2)
West (n = 200)	1 [Reference]
**Metropolitan statistical area**	
Nonmetropolitian area (n = 233)	0.9 (0.7–1.3)
Population <500,000 (n = 204)	1.2 (0.9–1.7)
Population ≥500,000 (n = 703)	1 [Reference]
**State plumbing code drinking fountains required per student^c^**	
1 fountain per <100 students (n = 182)	0.9 (0.7–1.3)
1 fountain per 100 students (n = 750)	1 [Reference]
1 fountain per >100 students (n = 146)	0.6 (0.4–0.9)
**State plumbing code allowance of nonplumbed water sources^d^**	
Nonplumbed water sources may substitute for all fountains (n = 135)	1.7 (1.1–2.6)
Nonplumbed water sources may substitute half of fountains (n = 647)	1.7 (1.3–2.2)
Nonplumbed water sources may not substitute for fountains (n = 315)	1 [Reference]

a Participants responded either “Yes, there are many”, “Yes, only a few”, “No, none work”, or “There are no water fountains” to the question “Does your school have working drinking fountains or dispensers that you can drink from?” “Widely available drinking fountains” correspond with participants reporting “Yes, there are many.”

b Odds ratios for age, sex, race/ethnicity, household income, census region, and metropolitan area size adjusted for one another; odds ratios for drinking fountains required per student and allowance of nonplumbed water sources adjusted for age, sex, race/ethnicity, household income, and metropolitan area size.

c Excluded participants in states where there was no code.

d Excluded participants in states where the state code did not specify a fountain to student ratio or a proportion of fountains that could be replaced by nonplumbed sources.

## Discussion

We found that more than 40% of youth reported that there are few working drinking fountains or dispensers available in their school, and 1 in 25 youth reported there are no working fountains or dispensers. We also found regional differences in youth-reported access, with reported access lowest in the West and highest in the Midwest. These differences may be partially due to state plumbing codes, which differ greatly by region. To our knowledge, this is the only study to examine school drinking water access at a national level or to examine the association of state plumbing codes with water access in schools.

Increasing free drinking water access in schools is gaining greater attention as a means of encouraging healthy beverage intake among children, with recent regulatory requirements implemented at both the state and federal level (9,14). Data on the availability of free drinking water in schools is limited. A 2011 national survey of school administrators found that approximately two-thirds of middle and high school students had drinking fountains available in the school cafeteria, approximately 80% had fountains available in gymnasium or locker room areas, approximately 98% had fountains available in hallways near classrooms, and approximately 40% had fountains in other school locations (15). On a local level, Patel et al reported in 2011 that only half of schools surveyed in the Bay Area of California had water available in food service areas before enactment of California Senate Bill 1413 and the national 2010 Healthy, Hunger-Free Kids Act, which both require schools to provide access to free drinking water during mealtimes in school food service areas (16). Regarding school wellness policies, Cradock et al found that school wellness policies in Massachusetts rarely covered access to drinking water (17). Although school administrators reported in 2011 that approximately half of middle and high school students were covered by written policies regarding availability of free drinking water (15), examination of actual district wellness policy documents found that approximately 85% did not include a written policy regarding availability of free drinking water throughout the day (18). Regarding water offered for sale in schools, data from the US Department of Agriculture’s School Nutrition Dietary Assessment IV suggest that bottled water is made available for sale through vending and a la cart sales during lunch in most middle and high schools (19).

Our study suggests that water access may be greater where plumbing codes require at least 1 drinking fountain per 100 students and where codes allow the substitution of nonplumbed water dispensers for plumbed drinking fountains. However, the clustering of these 2 attributes in state plumbing codes because of widespread adoption of published model codes makes it difficult to separate the effects of fountain to student ratio and allowing nonplumbed water fountains to be substituted for plumbed water fountains. Specifically, all state plumbing codes that specify fountain to student ratios of 1 fountain per more than 100 students also do not allow any substitution for plumbed drinking fountains. Although 3 non-Western states specify fountain to student ratios of 1 fountain per more than 100 students (Maine, Nebraska, and South Dakota), the small samples from these states limited our ability to separate the effect of plumbing codes on school water availability from that of region.

Regional factors other than plumbing codes could explain regional differences. In general, the arid climate of many Western states can create challenges in ensuring adequate drinking water supplies for their populations and may thus encourage an atmosphere of water conservation that overrides access concerns (20). Water quality problems may also affect the availability of tap water in schools, and regional differences exist in perceptions of tap water safety (21). The lowest level of access reported in our study occurred in the West and Northeast. Although systematic data on water quality at the point of use in schools are not available, problems such as agricultural contamination of drinking water in California’s Central Valley (22) and point-of-use drinking water contamination from old plumbing in Philadelphia schools (23) have been reported. However, undocumented problems with water quality in schools could exist in other places as well.

This study has several limitations. First, we could not separate the effects of plumbing codes from those of region. Second, we used youth-reported drinking fountain access, from a survey offered only in English, rather than objective measurement. Future research is needed on how student-reported water access corresponds to actual access and to validate water access survey items. Third, we did not have information on local municipal plumbing codes or school wellness policies, which may go beyond state plumbing codes in specifying water access requirements in schools. Fourth, we did not have data on the age of the school facilities. Age of school facilities may be a factor in how plumbing codes associate with actual water access, because codes may change over time and broken fountains may come out of service and not be replaced. Furthermore, water access may change over time as the number of students attending a school changes and schools undergo additions or renovations that either add or remove fountains. Finally, because of selection bias associated with the use of a convenience sample from a mail panel survey and a low response rate, our findings may not be generalizable nationally. However, the study population included participants from 47 states and the District of Columbia who were sampled proportionately to state populations, and participants were weighted to match the US Current Population Survey (12). A previous study has shown that prevalence estimates of certain items from HealthStyles (eg, health conditions and behaviors) are comparable to findings in the Behavioral Risk Factor Surveillance System, which uses a probability sampling technique (24).

We found that 4 in 10 youth report that there are only a few working drinking fountains or dispensers in their schools, and 1 in 25 indicated that they had no working fountains or dispensers in their school. Additionally, student-reported drinking fountain availability, after controlling for other factors, was significantly different by race/ethnicity, region, the fountain to student ratio specified in state plumbing codes, and whether the plumbing code allowed nonplumbed water sources. Further research regarding how drinking fountain requirements in plumbing codes relate to drinking water availability in schools and other places would be useful for states and communities that wish to increase drinking water access to support healthy beverage intake among their residents.
